# Wait Time from Suspicion to Surgery for Breast Cancer in Manitoba

**DOI:** 10.7759/cureus.680

**Published:** 2016-07-11

**Authors:** Maged Nashed, Tara Carpenter-Kellett, Pascal Lambert, Grace Musto, Donna Turner, Andrew Cooke

**Affiliations:** 1 Radiation Oncology, CancerCare Manitoba; 2 Community Oncology, CancerCare Manitoba; 3 Epidemiology and Cancer Registry, CancerCare Manitoba

**Keywords:** breast cancer wait suspicion surgery manitoba

## Abstract

Introduction:

Breast cancer (BC) is the most common cancer in women. The pathway for its diagnosis and treatment is relatively standardized. Nevertheless, there can be significant delays affecting the journey. The aim of this retrospective study is to describe the BC wait times (WT) from suspicion to first surgery in Manitoba and to examine factors associated with WT variability.

Methods:

The cohort is composed of patients with stages I-III breast cancer who were diagnosed between September 1, 2009, and August 31, 2010, and referred to a cancer center. Patients’ journeys were tracked and divided into three sequential intervals from suspicion to first diagnostic test, from first diagnostic test to diagnosis and from diagnosis to first surgery.

Results:

Four hundred and four patients were included of whom 134 presented through the screening program. There was no difference between the study cohort and population data from the provincial Cancer Registry concerning the distribution of age, stage of cancer or residence. The median WT from suspicion to surgery was 78 days.

In the screen-detected group (SD), a difference in median WT from suspicion to first diagnostic test was found for distance. This finding was first to test location, where those who travel less had longer WT than those who have longer journeys. Patients who went to centers that offer both imaging and biopsy services, even if the required test is imaging only, had to wait longer than those who went to centers that provide imaging only. SD patients needing more than one diagnostic test had a longer WT from the first test to pathological diagnosis if the first test did not include a biopsy.

Patients who were seen by surgeons before final pathological diagnosis had a shorter WT from diagnosis to first surgery than those who had the surgical consult after tissue diagnosis was made. A delay to surgery was observed in the whole cohort if a plastic surgeon is required in addition to the surgical oncologist and the non-screen detected group if a radiologist is necessary.

Conclusions:

Variability in WT from suspicion to surgical management was found between various BC patient groups and between diagnostic centers with different types of services. The order of the provided diagnostic and surgical services may have contributed to WT. Addressing this variability by restructuring the care pathway and improving communication between different disciplines, has the potential to reduce WT.

## Introduction

Breast cancer (BC) is the most common cancer in women. An estimated 860 women developed BC in Manitoba out of 25,000 in Canada in 2015. An estimated 200 deaths from BC have occurred in Manitoba out of 5,000 deaths in Canada in 2015 [1].

The care pathway for BC contains a series of events starting from first clinical or mammographic suspicion to the completion of all adjuvant therapies. The pathway for diagnosis and treatment of BC is relatively standardized for the majority of patients. Nevertheless, there can be significant delays affecting different parts of the journey.

Various studies have shown an adverse impact of delay to surgery on cancer-specific mortality [2-5]. However, others have not noted a link between delayed referral and survival of BC patients [6]. Regardless of the clinical impact of delays on patients’ outcome, the psychological burden suffered by patients while waiting for investigations and treatment is immense [7-10].

In the United Kingdom, a plan to cut wait times (WT) was implemented in 2000. In the 2014-2015 annual report, 93.3% of urgently referred symptomatic breast patients were seen by a specialist within 14 days, and 95.9% of them received their first treatment within 62 days from referral. 98.8% of BC patients received their first treatment within 31 days of diagnosis [11].

In Canada, acceptable WT from the abnormal screening mammogram to diagnosis is seven weeks if a biopsy is performed [12]. However, there is no guidance on acceptable WT from suspicion to diagnosis in the non-screen detected cohort [12]. Moreover, there is no Pan-Canadian benchmark for an acceptable WT from diagnosis to cancer surgery. However, certain jurisdictions such as Ontario aims for a typical patient with invasive breast Cancer to receive definitive surgery within 28 days from the decision to treat made [13]. In Nova Scotia, the median number of days from BC detection to referral to an oncology center increased from 53 to 61 days from 2000 to 2004 [14]. In Alberta [15], the median waiting time from BC diagnosis to surgery increased from 14 days in 1997 to 20 days in 2000. The median WT from diagnosis to definitive surgery in Ontario for patients diagnosed between 1995-2003 was 17 days [16] while the median WT from the first abnormal imaging to definitive surgery was 52 days [17].  

In Manitoba, the radiation treatment WT improved significantly from 2001 to 2005 [18]. However, the overall time from suspicion of completing adjuvant treatment lengthened because the median time from diagnosis to surgery increased from 22 to 35 days negating the reduction seen in radiation treatment WT. This fact demonstrates that all intervals in the care pathway need to be considered.

The aim of this retrospective study is to describe the WT from suspicion to first treatment for BC in Manitoba and to examine factors that may be associated with WT variability. The Manitoba government has announced a $40 million initiative aimed at reducing patient cancer WT [19]. The outcome of our study will help guide this effort. 

## Materials and methods

The study cohort is composed of patients who were part of a planned audit designed to explore the status of WT for all BC patients diagnosed between September 1, 2009, and August 31, 2010, and referred to Cancer Care Manitoba (CCMB) for consideration of adjuvant treatment. The approval from the University Of Manitoba Health Research Ethics Board was obtained, and patient consent was waived. The patients presented either through a primary care provider’s office or through the provincial screening program which screens women between 50-74 years. Patients with stages 0 and IV were omitted from the study cohort since their care pathway does not follow the routine steps that are commonly followed in stages I-III.

Patients were tracked from the time of the first presentation to the time of first surgery, and WTs were divided into three sequential intervals calculated in calendar days. 1- Suspicion to first diagnostic test. The suspicion date is the date of the abnormal screening mammogram for the screen-detected group (SD) or the date of the last visit to the primary care provider that preceded the first diagnostic test for the non-screen detected group (NSD). The first diagnostic test date is the date of the first diagnostic radiology or tissue-obtaining procedure done after the suspicion date. 2- First diagnostic test to diagnosis. Diagnosis date is the date of the first confirmatory BC pathology report. 3- Diagnosis to the first surgery. The first surgery is defined as different variants of mastectomy, lumpectomy or incisional/excisional biopsy (when not followed by another surgical procedure).

Data collection:

Data was abstracted from original radiologic, surgical, and pathological reports contained in the patients’ charts at diagnostic and treatment centers and also from CCMB records. For missing data, especially the suspicion date, attempts were made to contact the referring physician’s office. Data collected included patient demographics, dates, and types of investigations leading to diagnosis, date of the pathology report and date of first surgery. Cancer staging manual (TNM, American Joint Committee on Cancer 2002) was used to determine anatomic BC stages. The Urban dwelling was identified as those who reside in one of the two principal cities in the province.

Statistical analysis:

Quantile regression was used to calculate median differences between subgroups of interest for the three intervals of WT. Predictors for the first interval included cancer stage, distance from the patient origin to first test location, age, and first test type. Predictors for the second interval included cancer stage, age, the number of times tested, and first test type. Predictors for the third interval included the type of surgery, whether the first consult was before or after the pathological diagnosis, screening status, urban/rural residence, and distance from residence to surgeon location. The continuous variables of distance and age were analyzed using restricted cubic splines with three knots (at 0.10, 0.50, and 0.90). The distance between residence and first test location was calculated with the Haversine formula using latitude and longitude information from postal codes. First test type was categorized using two separate approaches: the test types available at the first test location and the actual types of tests administered for that first test. Data was analyzed using STATA version 11.2.

## Results

Four hundred and four patients with stages I-III BC were included (Table 1). The SD group represents one-third of the study cohort. There were only two patients < 50 years old in the SD group because screening is offered only to women ≥50 with rare exceptions. 25.5% of the NSD patients were under 50 years of age. Patients with stage I represent one and two-thirds of the NSD and SD groups, respectively.

**Table 1:** Description of the study cohort. *Urban dwelling in the 2 major cities in the province was identified by postal codes.

		Non-screened		Screened		Total	
		(N = 270)		(N = 134)		(N = 404)	
		N	%	N	%	N	%
Age groups	<50	69	25.6	2	1.5	71	17.6
	≥50	201	74.4	132	98.5	333	82.4
Residence	Urban *	192	71.1	87	64.9	279	69.1
	Rural	77	28.5	47	35.1	124	30.7
	Unassigned	1	0.4	0	0	1	0.2
Stage of cancer	I	97	35.9	89	66.4	186	46
	II	125	46.3	37	27.6	162	40.1
	III	48	17.8	8	6	56	13.9
Service provided at first test location	FNA only	9	3.3	2	1.5	11	2.7
	Imaging only	187	69.3	36	26.9	223	55.2
	Imaging and biopsy	74	27.4	96	71.6	170	42.1
First test type	Biopsy	13	4.8	2	1.5	15	3.7
	Imaging	253	93.7	59	44	312	77.2
	Imaging and biopsy	4	1.5	73	54.5	77	19.1
Number of diagnostic tests	1	4	1.5	53	39.6	57	14.1
	2	184	68.1	48	35.8	232	57.4
	3	82	30.4	33	24.6	115	28.5
Surgical consult	Before diagnosis	78	29.2	6	4.5	84	20.9
	After diagnosis	189	70.8	128	95.5	317	79.1
Service required in addition to breast surgeon for first surgery	None	193	72.3	75	56	268	66.8
	Radiologist	42	15.7	55	41	97	24.2
	Plastic surgeon	32	12	4	3	36	9

Population data from the provincial Cancer Registry was used to study if our cohort is a representative sample of patients with BC diagnosed in Manitoba during the same period. The study group represents 53.3% (404/758) of patients diagnosed with the same stage of BC during the same period. There was no difference between the two groups concerning the distribution of age, stage of cancer or residence (Table 2).

**Table 2:** Comparison of the study cohort with provincial Cancer Registry data.

Characteristic	Parameter	Cancer Registry	Study cohort	*p*
		No. (%)	No. (%)	
Stage	I	341 (44.9)	186 (46.04)	0.739
	II	299 (39.45)	162 (40.1)	
	III	118 (15.57)	56 (13.86)	
Age	<50	137 (18.07)	71 (17.57)	0.832
	≥50	621 (81.9)	333 (82.43)	
Residence	Rural	256 (33.77)	124 (30.69)	0.244
	Urban	490 (64.64)	279 (69.06)	
	Unassigned	12 (1.58)	1 (0.2)	

**Figure 1:**
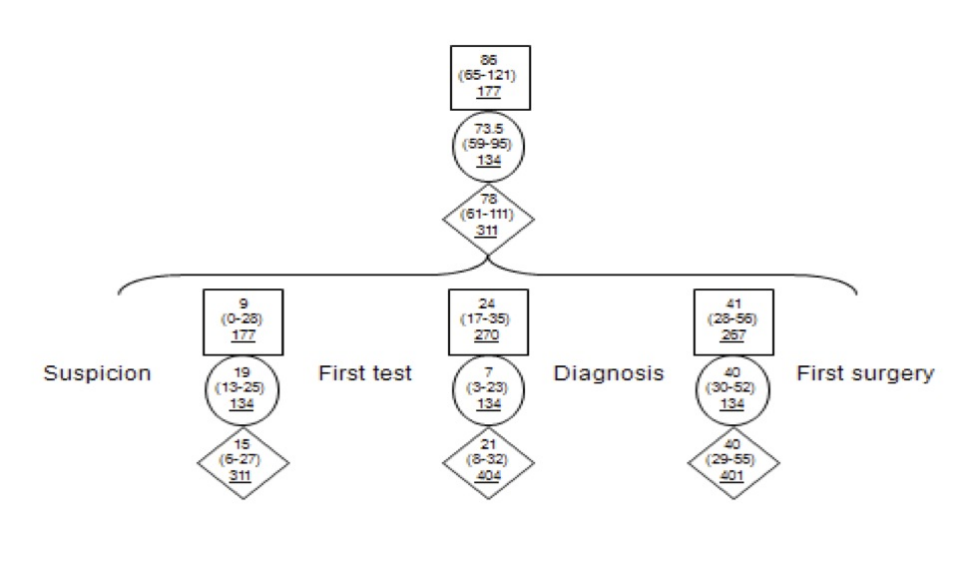
Median WT in days (25th and 75th percentiles) for all patients (diamonds), SD group (circles) and NSD group (rectangles). Number of observations in each subgroup is underlined.

The overall WT and the three intervals WTs for all patients from suspicion to first surgery are shown in figure 1. The median WT from suspicion to surgery for the whole cohort was 78 days. Despite differences in median WT between SD and NSD groups from suspicion to first diagnostic test and from first diagnostic test to diagnosis, both groups had similar median WT in the third interval (diagnosis to first surgery).

**Figure 2:**
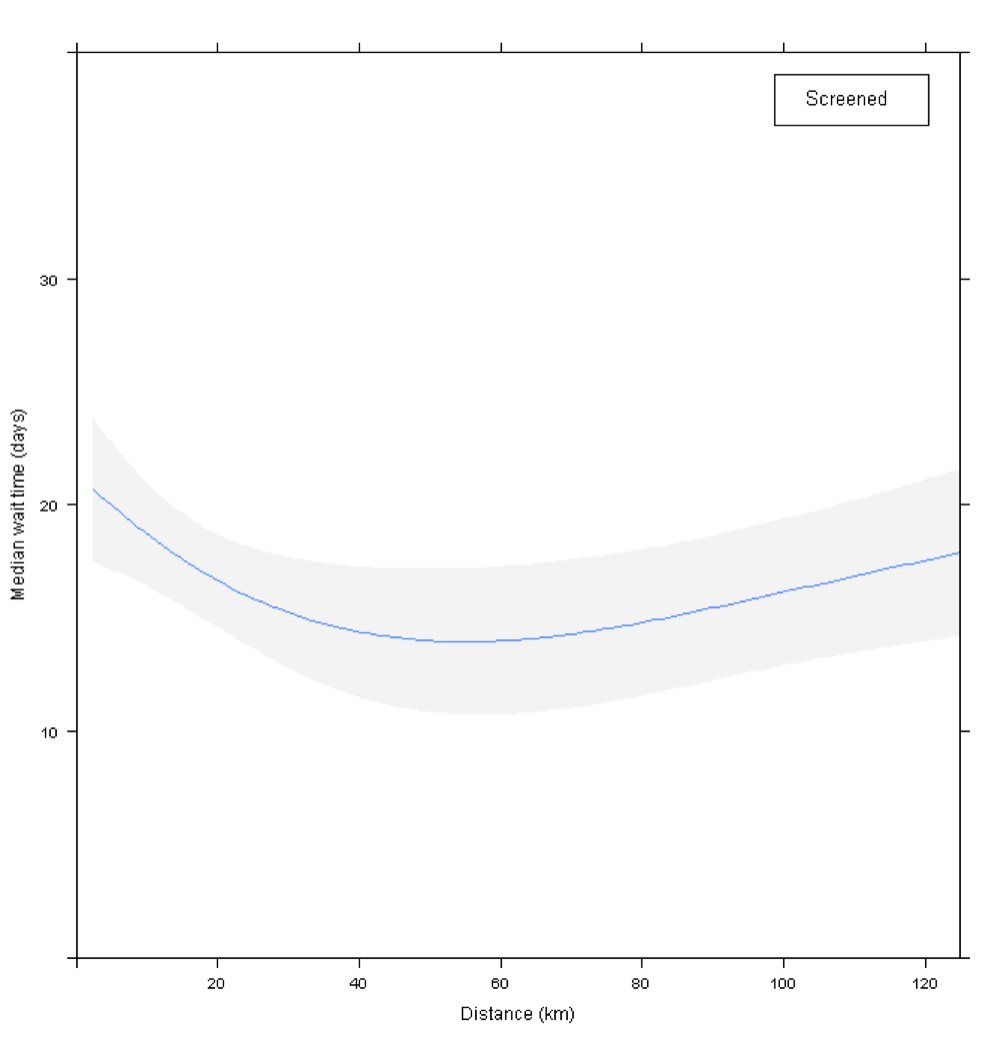
Effect of distance from patient’s residence to first test center in the SD group on median WT from suspicion to first diagnostic test. Shaded area represents 95% confidence limits.

In the interval from suspicion to first diagnostic test, no direct comparison could be performed between the SD and NSD groups because of different entry points to the care pathway. For the NSD group, no patient variables were associated with longer WT. Within the SD group (Table 3) difference in median WT was found for distance to first test location (*p* <0.001). Those with a distance of under 15 km representing 64% of the cohort and almost exclusively of urban residence had a WT of about 20 days. WT for those who travel distance of 15 km (almost entirely of rural residence) decreased to about 15 days at about 50 km (Figure 2). Also in the SD group (Table 3), patients who went to centers that offer both imaging and biopsy services, even if the required test was imaging only, had to wait longer than those who went to centers that provide imaging only (*p *= 0.002).

**Table 3:** Predictors of longer WT for the intervals from suspicion to first diagnostic test and from first test to diagnosis.

				Median difference (days)	95% CI	*p*
From suspicion to first diagnostic test (SD group)						
Intercept				13.18	10.31 - 16.05	<0.001
Distance to first test location						
'				-0.02	-0.14 - 0.1	<0.001
''				0.44	-0.41 - 1.28	
Service provided at first test center						
Type of first test performed		Service offered at diagnostic location				
Imaging		Imaging		(Reference^ a^)		
Imaging + Biopsy		Imaging + Biopsy		7.05	4.24 - 9.87	<0.001
Imaging		Imaging + Biopsy		5.94	2.24 - 9.65	0.002
^ a Imaging as the first test in a service location that offers imaging only has an expected median wait time of 12.9 days when patients are at a distance of 15 km from the testing location^						
From first test to pathological diagnosis						
Intercept				4	0.71 - 7.29	0.017
Diagnostic tests performed						
Group	Type of 1^st^ test		Number of tests			
SD	Imaging + biopsy		1 test	(Reference^ b^)		
SD	Imaging		2 tests	22	16.65 - 27.35	<0.001
SD	Imaging		3 tests	15	9.43 - 20.57	<0.001
SD	Imaging + biopsy		2 tests	-1	-7.38 - 5.38	0.758
SD	Imaging + biopsy		3 tests	1	-8.62 - 10.62	0.838
NSD	Imaging		2 tests	20	16.26 - 23.74	<.001
NSD	Imaging		3 tests	25	20.71 - 29.29	<.001
NSD	Imaging + biopsy		1 test	-1	-4.29 - 2.29	0.551
^b Imaging and biopsy as the first and only test has an expected median wait time of 4 days^						

In the interval from the first test to pathological diagnosis (Table 3), SD patients who underwent only one test that included a biopsy were used as a reference group as they are expected to be the fastest group to obtain a tissue diagnosis. SD patients needing more than one test had a longer WT if the first test did not include a biopsy (*p *< 0.001). A similar pattern was observed in the NSD patients (*p *< 0.001), although there was a small number of patients in this subgroup who underwent one test only. This behavior is expected since most of the patients in the NSD group have to get a diagnostic mammogram before a biopsy.

In the final interval from diagnosis to first surgery (Table 4), within the NSD group, those who were seen by surgeons prior to final pathological diagnosis i.e., patients with a high level of clinical suspicion of malignancy, had a shorter WT to surgery (*p = *0.001) than those who had the surgical consult after tissue diagnosis was made. A delay in the median time to surgery was observed in the whole cohort (*p = *0.036) and also when SD and NSD groups are examined separately if a plastic surgeon was required in addition to the surgical oncologist. In the NSD group, if a radiologist was required (e.g. to perform wire localization), a delay in WT to surgery was observed (*p *= 0.036).

**Table 4:** Predictors of longer WT in the interval from diagnosis to first surgery.

				Median difference (days)	95% CI	*p*
Order of surgical consult						
-Entire cohort						
Intercept				42	39.72 - 44.28	<0.001
Order of surgical consult			After diagnosis	Reference^ a^		
			Before diagnosis	-8	-12.98 - -3.02	0.002
^a After diagnosis has an expected median wait time of 42 days^						
-SD group						
Intercept				40	36.22 - 43.78	<0.001
Order of surgical consult	After diagnosis			Reference^ b^		
	Before diagnosis			-6	-22.78 - 10.78	0.481
^b After diagnosis has an expected median wait time of 40 days^						
-NSD group						
Intercept				42	39.58 – 44.42	<0.001
Order of surgical consult		After diagnosis		Reference^ c^		
		Before diagnosis		-8	-12.53 - -3.47	0.001
^ c After diagnosis has an expected median time of 42 days^						
Service required for first surgery						
-Entire cohort						
Intercept				37	33.76 - 40.24	<0.001
Service required		Surgical oncologist		Reference^ d^		
		+ radiologist		5	-1.18 – 11.18	0.112
		+ plastic surgeon		10	0.68 – 19.32	0.036
^d Only breast surgeon has an expected median wait time of 37 days^						
-SD group						
Intercept				38	32.01 – 43.99	<0.001
Service required		Surgical oncologist		Reference^ e^		
		+ radiologist		2	-7.14 – 11.14	0.666
		+ plastic surgeon		35	11.44 – 58.56	0.004
^e Only breast surgeon has an expected median wait time of 38 days^						
-NSD group						
Intercept				36	32.84 – 39.16	<0.001
Service required		Surgical oncologist		Reference^ f^		
		+ radiologist		8	0.54 – 15.46	0.036
		+ plastic surgeon		10	1.67 – 18.33	0.019
^f Only breast surgeon has an expected median wait time of 36 days^						

## Discussion

Wait times for the diagnosis and management of cancer continues to be a hot topic for Canadians [14 - 18]. Identifying factors that are associated with variability in WT could provide an opportunity to reduce long waits to get treatment and possibly impact the prognosis for women with breast cancer. The province of Manitoba has announced the InSixity initiative designed to reduce cancer journey time from suspicion to first treatment to within 60 days. In order to invest wisely to achieve this goal, it is crucial to identify bottlenecks in the care pathway and work to eliminate them.

In this retrospective study, we report on the WT from suspicion to first surgery for patients with BC diagnosed in Manitoba between September 2009 and August 2010. Our data are representative of the Manitoba population since there was no difference between our cohort and the provincial Cancer Registry concerning age, stage of cancer or residency (Table 2).  Also, our results are similar to unpublished administrative data from the provincial Cancer Registry that showed a very similar median WT for BC patients (from suspicion to first treatment) in 2013 at 77.5 days (compared to 78 days in this study).

Our data suggest that current organization of centrally located diagnostic services may contribute to a prolongation in WT from suspicion to the first test in the SD group (Table 3). First, the urban patients had a WT longer than for rural patients, which implies that distance itself was not the determining factor for WT. Second, there was a delay in obtaining the first test for patients who went to diagnostic centers that offered a broad scope of diagnostic tests (imaging and biopsy in this case) even if the required test was imaging only. Therefore, it is not the type of the test that affects WT but rather the ability of the test center in managing the required services. Consequently, improving the organization of the centrally located diagnostic facilities may contribute to a reduction of the WT.

The longer median WT from the first test to pathological diagnosis in the NSD group (24 days) compared to SD group (7 days) is likely related to the need to perform a diagnostic mammogram after the clinical suspicion. Another contributing factor to the shorter WT in SD group is that patients are directly referred from radiology to where a biopsy is performed.  Therefore, restructuring the care pathway for the NSD group by allowing a similar referral pattern to SD group may lead to a reduction of WT for this subgroup.

In both SD and NSD groups, patients who had to image only as the first test had a longer interval to pathological diagnosis than those who had a biopsy as a part of the first diagnostic test (Table 3). Commonly patients with larger cancers get a biopsy early which may explain our findings. Nevertheless, our results highlight the need to perform a biopsy as soon as possible.

The median WT from diagnosis to surgery was 21 days in Nova Scotia in 2003-2004 [14] and 20 days in Alberta in 2000 [15]. In Ontario, the median WT was 17 days in the period between 1995 and 2003 [16]. These remarkable results are far better than the measured median wait time in our cohort (40 days). However, one has to note that the median WT in Ontario showed a progressive and lengthening from 1995-1999 to 2000-2003. Also, there was no definition given for the diagnosis date, which could be interpreted as the date of biopsy performed, pathology issued or date of review by the specialist.

The long WT from diagnosis to surgery in our cohort (40 days) highlights the need for re-structuring the care pathway. First, patients in the NSD group who were seen by surgical oncologists before final tissue diagnosis had a shorter median WT compared to those who were seen after pathological diagnosis was made (Table 3). Lack of this observation in the SD cohort may be related to the small number of patients (six) who were seen by the surgical oncologist before pathological diagnosis being made. Therefore, expanding this approach for patients with a high level of clinical suspicion of malignancy could potentially reduce WT to surgery. Second, if a plastic surgeon was required in addition to the surgical oncologist, patients had to wait longer (median ten days) than those who did not require a plastic surgeon. A similar finding was reported in Ontario where 15 days are added to the median WT if a preoperative consultation to plastic surgery was required [17]. This could potentially be remedied by improving communication and coordination between different disciplines.

This retrospective study may be limited to the missing data. For example, the suspicion date was missing in 97 patients in the NSD group. Moreover, we analyzed only cases referred to CCMB within a defined timeframe. Therefore, we did not perform a population-based analysis. However, we have shown that our cohort does not differ from the general population about age, stage of cancer or residence (Table 2). The data reported in this work may not be generalizable to the current Manitoba population if systemic changes have occurred since the study cohort was diagnosed. Also, the results of this work do not apply to patients with non-invasive cancer or those with stage IV BC since these groups were not included in the study cohort.

While the results of the present study suggest diagnostic and surgical delays may contribute to longer WT, we did not assess variables that may have influenced the overall care times such as some staging investigations, procedure complication rates, and missed or canceled appointments. Also, we did not address the effect of different workloads between diagnostic or surgical centers on WT. A separate research project needs to be dedicated to addressing this particular issue.

## Conclusions

Variability in WT to diagnosis and surgical treatment was found between various BC patient groups and between diagnostic centers with different types of services. The order of the provided diagnostic and surgical services may have also contributed to longer WT. Addressing this variability has the potential to reduce WT for breast cancer patients from suspicion to first surgery. This could be achieved by restructuring the care pathway and improving communication between different disciplines.
